# Supplementary material for: An integrated multi-omics analysis of sleep-disordered breathing traits implicates P2XR4 purinergic signaling
Source: Commun Biol. 2023 Jan 31;6:125. doi: 10.1038/s42003-023-04520-y (PMC9889381; doi:10.1038/s42003-023-04520-y)
Supplement: Supplementary file 2 — Supplementary Materials [file 42003_2023_4520_MOESM2_ESM.pdf]

An integrated multi-omics analysis of sleep-disordered breathing traits implicates P2XR4 purinergic signaling

Kurniansyah et al.

Supplementary Note 2
The Multi-Ethnic Study of Atherosclerosis (MESA) 2
The Hispanic Community Health Study/Study of Latinos (HCHS/SOL) 2
Genotyping and imputation in HCHS/SOL 3
The Women's Health Initiative (WHI) 3
RNA sequencing in WHI 4
Secondary analysis phenotypes 5
Supplementary Figures 6
Supplementary Figure 1. Heatmap of estimated log-fold gene expression change with SDB phenotypes across tissues without BMI adjustment in MESA 6
Supplementary Figure 2. Heatmap of estimated log-fold gene expression change with SDB phenotypes across tissues from BMI-adjusted analysis in MESA 7
Supplementary Figure 3. Spearman correlations between estimated log-fold changes in gene expression across SDB phenotypes and tissues in BMI adjusted analysis in MESA (top genes) 8
Supplementary Figure 4. Correlation between the SDB phenotypes 9
Supplementary Figure 5. Spearman correlations between estimated log-fold changes in gene expression across SDB phenotypes and tissues in analysis without BMI adjustment (all genes). 10
Supplementary Figure 6: Spearman correlations between estimated log-fold changes in gene expression across SDB phenotypes and tissues in analysis BMI adjustment (all genes). 11
Supplementary Figure 7. Comparison of the associations between monocyte-based tPRSs and whole-blood gene expression in WHI 12
Supplementary Figure 8. MESA data flow across various measures in the 1st and 5th exam, and the sleep ancillary study. 13
Supplementary Figure 9. HCHS/SOL data flow across genotyping, metabolomics, and sleep data. 14
Supplementary References 14

Supplementary Note

The Multi-Ethnic Study of Atherosclerosis (MESA)

MESA is a longitudinal cohort study [1], established in 2000, that prospectively collected risk factors for development of subclinical and clinical cardiovascular disease among participants in six field centers across the United States (Baltimore City and Baltimore County, MD; Chicago, IL; Forsyth County, NC; Los Angeles County, CA; Northern Manhattan and the Bronx, NY; and St. Paul, MN). The 1st and 5th MESA exams took place between 2000-2002, and 2010-2012, respectively, and whole blood was drawn from participants in both exams. For about 1,400 participants, blood was used later for RNA extraction and/or proteomics in at least one of the exams. In addition, a sleep study ancillary to MESA occurred shortly after MESA exam 5 during 2010-2013. Sleep study participants underwent single night in-home polysomnography (Compumedics Somte Systems, Abbotsville, Australia, AU), as previously described [2]. The number of individuals with each type of data and at each time point (exam 1 and exam 5) varies. **Figure S1** in the Supplementary Information visualizes the data flow and overlaps across the various measures used in this study: whole-genome genotyping, RNA-seq, and sleep. The study was approved by Institutional Review Boards in all study centers and participants provided written informed consent.

The Hispanic Community Health Study/Study of Latinos (HCHS/SOL)

The HCHS/SOL is a longitudinal cohort study of U.S. Hispanics/Latinos [3,4] recruited from four geographic regions: Bronx NY, Chicago IL, Miami FL, and San Diego CA. The HCHS/SOL baseline exam occurred on 2008-2011, where 16,415 participants were enrolled via multi-stage probability sampling. HCHS/SOL individuals who consented further participated in an in-home sleep study, using a validated type 3 home sleep apnea test recording airflow (via nasal

pressure), oximetry, position, and snoring (ARES Unicorder 5.2; B-Alert). Genetic data were measured and imputed to the TOPMed freeze 5b reference panel as previously described, for individuals who consented at baseline [5,6]. More information about genotyping and imputation is provided in the Supplementary Information. Metabolomic data were also measured for n=~4,000 individuals selected at random out of those with genetic data [7]. **Figure S2** in the Supplementary Information provides the data flow in HCHS/SOL, focusing on individuals with genetic data and wide consent for genetic data sharing. All participants provided written informed consent at their recruitment site and the study was approved by the institutional review boards at all participating institutions.

Genotyping and imputation in HCHS/SOL

Blood was drawn from HCHS/SOL participants during the baseline exam. Individuals who consented to genetic studies were genotyped using an Illumina Omni2.5M array, which included 150,000 custom-selected Single Nucleotide Polymorphisms (SNPs) including ancestry-informative and Amerindian-specific variants. Global ancestry proportions measuring the proportion of the genome inherited from European, African, and Amerindian ancestors and genetic principal components were computed as previously reported [5]. The genotypes were imputed to the Trans-Omics in Precision Medicine (TOPMed) freeze 5b reference panel as described in [6].

The Women's Health Initiative (WHI)

The WHI is a prospective national health study focused on identifying optimal strategies for preventing chronic diseases that are the major causes of death and disability in postmenopausal women [8]. The WHI initially recruited 161,808 women between 1993 and 1997 with the goal of including a socio-demographically diverse population with racial/ethnic minority groups proportionate to the total minority population of US women aged 50-79 years. The WHI consists of two major parts: a set of randomized Clinical Trials and an Observational Study. The

WHI Clinical Trials (CT; N=68,132) includes three overlapping components, each a randomized controlled comparison: the Hormone Therapy Trials (HT), Dietary Modification Trial, and Calcium and Vitamin D Trial. A parallel prospective observational study (OS; N = 93,676) examined biomarkers and risk factors associated with various chronic diseases. While the HT trials ended in the mid-2000s, active follow up of the WHI-CT and WHI-OS cohorts has continued for over 25 years with the accumulation of large numbers of diverse clinical outcomes, risk factor measurements, medication use, and many other types of data.
A total of 11,071 WHI participants have whole-genome sequencing data via TOPMed, and 1,274 of these participants have RNA-seq measured in venous blood via TOPMed.

RNA sequencing in WHI

RNA-seq was performed via the Trans-Omics in Precision Medicine (TOPMed) program. The WHI RNA samples (N=1,335) were collected from Long Life Study (LLS) participants as part of the LLS Blood Protocol using the PreAnalytiX PAXgene blood tubes, a collection system designed to preserve RNA from whole blood. After collection in participant's homes throughout the US, PAXgene tubes were the last of five tubes drawn from each participant, mixed carefully (inverted 8-10 times), kept at room temperature for a minimum of 2 hours post draw, and shipped overnight with cool packs to the Fred Hutch Specimen Processing Lab (SPL). Upon receipt at the SPL, PAXgene tubes were stored at -80 degrees C until they could be transferred to the Fred Hutch Public Health Sciences Biomarker Lab, where the vials were kept frozen at -80 degrees C. Within about a month of collection, the lab extracted total RNA, including miRNA, using the PreAnalytiX method (*PAXgene Blood miRNA Kit Handbook, Qiagen, 05/2009)* designed for use with the PAXgene blood collection tubes. A qualitative assessment by agarose gel electrophoresis of RNA integrity was done at the time of extraction. The RNA was quantified by NanoDrop. The elution volume of 76 µL of extracted RNA was divided between two RNA 'Parent' vials without further dilution, frozen at -80 degrees C, and shipped overnight on dry ice

to the WHI biorepository for long-term storage at -80 degrees C. RNA sequencing for WHI was performed at the Broad Institute using the unified TOPMed protocols. More information about RNA sequencing protocols in TOPMed is available here https://github.com/broadinstitute/gtex-pipeline/blob/master/TOPMed_RNAseq_pipeline.md.

Secondary analysis phenotypes

In secondary analysis, we adjusted the SDB phenotypes-RNAseq associations to cardiometabolic and glycemic measures with evidence of causal effect on SDB, including pulse pressure (PP), type 2 diabetes, waist-to-hip ratio, glycated hemoglobin (HbA1c), and for alcohol use. Blood Pressure (BP) was measured three times at 1-minute intervals after a 5-minute initial rest using a Dinamap PRO 100 automated oscillometric device (Critikon, Tampa, FL) with the participant seated. The average of the second and third BP measurements was used in the analysis. For individuals reporting the use of antihypertensive medications, we raised SBP and DBP values by 15 mmHg and 10 mmHg, respectively [9]. PP was calculated as SBP minus DBP. Type 2 Diabetes was defined by fasting glucose criteria based on 2003 American Diabetes Association. Individuals who self-reported taking insulin or hypoglycemic medications were categorized as having type 2 diabetes. Waist-to-hip ratio was calculated as waist circumference divided by hip circumference. HbA1c was measured using an assay serum Tosoh G7 instrument. Alcohol use was defined by self-reported current drinking status.

Supplementary Figures

Supplementary Figure 1. Heatmap of estimated log-fold gene expression change with SDB phenotypes across tissues without BMI adjustment in MESA

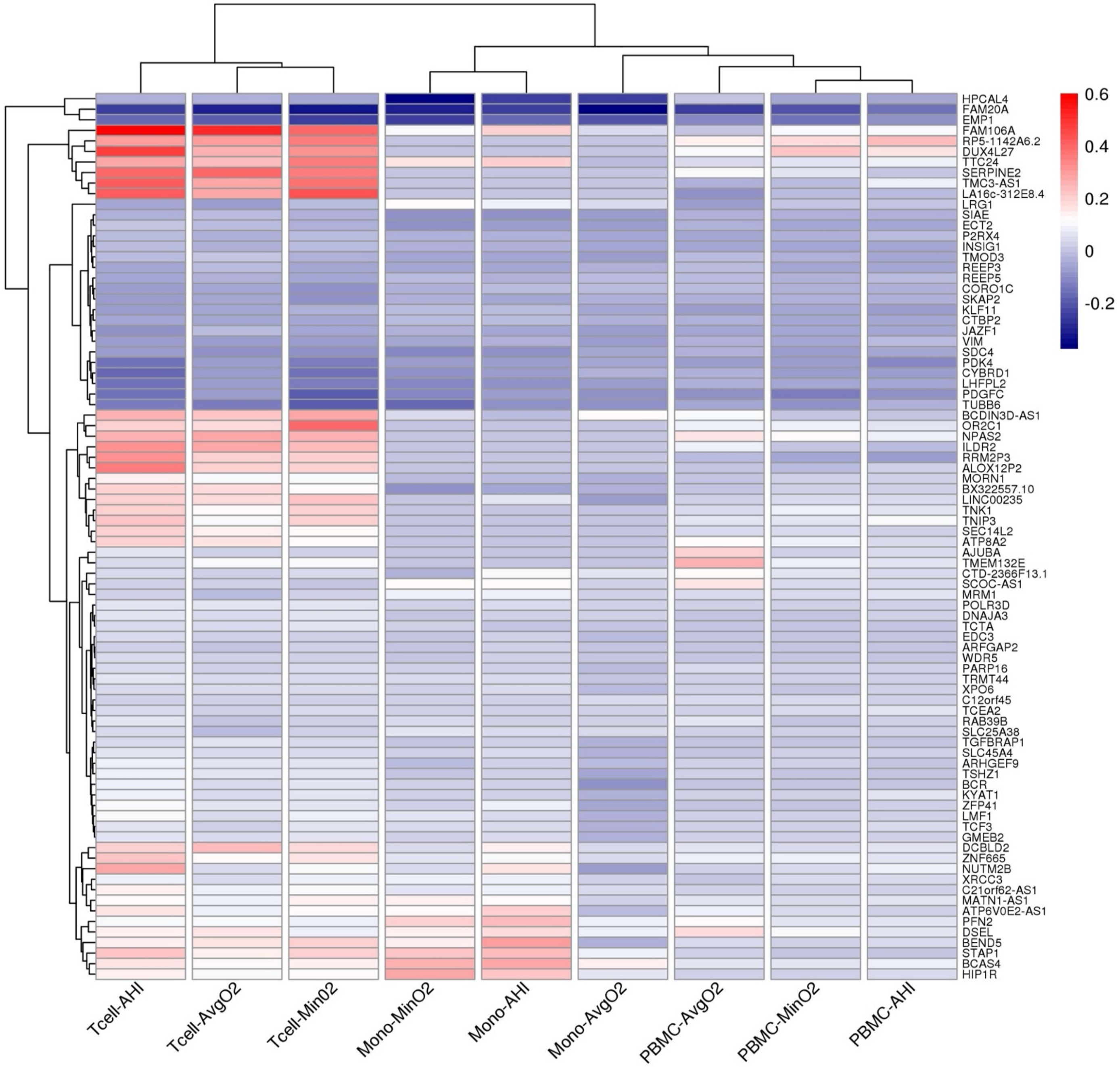

Genes displayed in this figure are those that had FDR p-value<0.1 in association analysis without BMI adjustment. FDR adjustment was computed separately in each set of associations defined by cell type and SDB phenotype. Red color indicates increased expression with improved SDB phenotypes, while purple colors indicates decreased expression with improved SDB phenotypes. Note that AHI effect estimates were oriented to match the directions of AvgO2 and MinO2.

Supplementary Figure 2. Heatmap of estimated log-fold gene expression change with SDB phenotypes across tissues from BMI-adjusted analysis in MESA

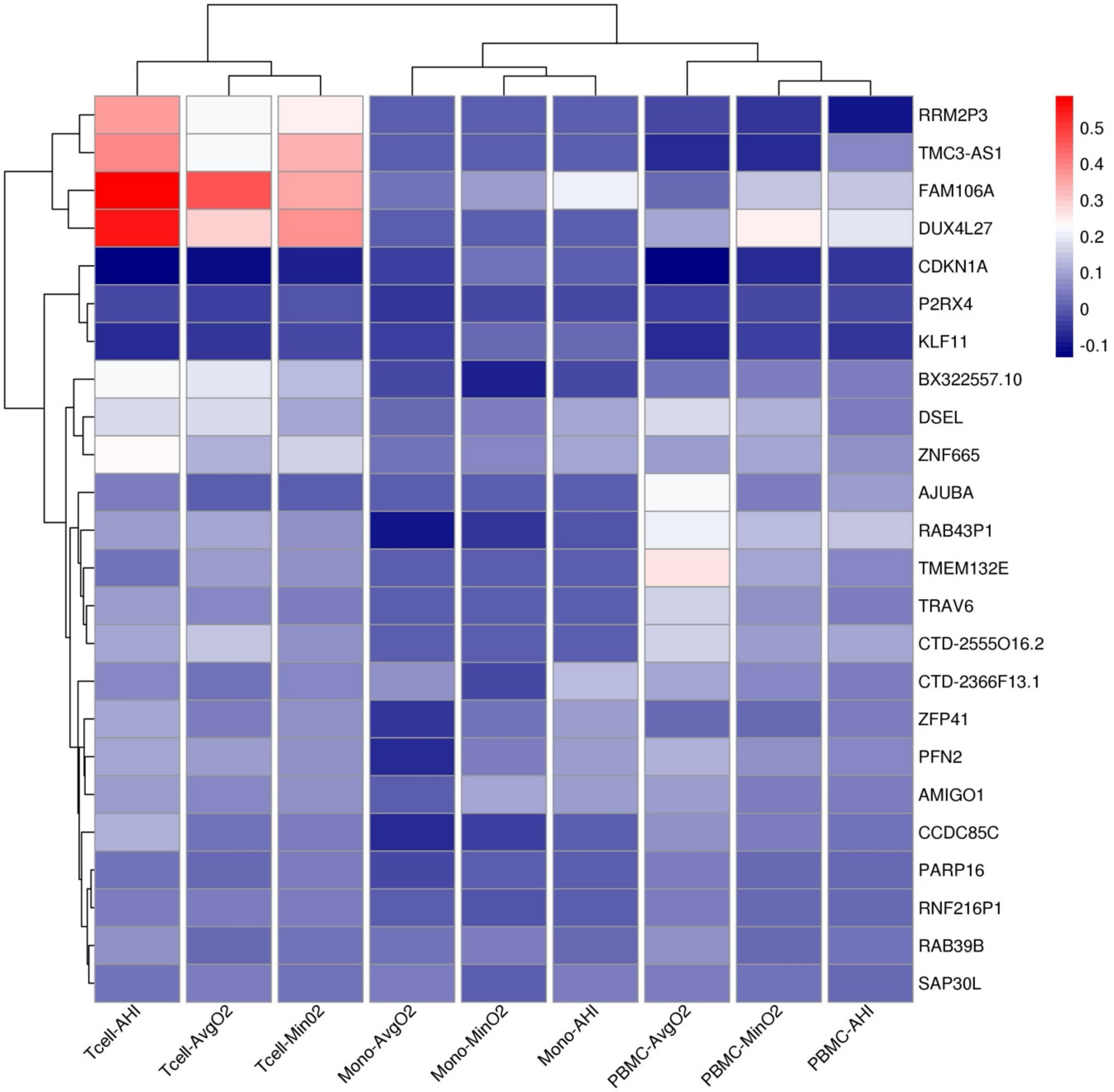

Genes displayed in this figure are those that had FDR p-value<0.1 in association analysis with BMI adjustment. FDR adjustment was computed separately in each set of associations defined by cell type and SDB phenotype. Red color indicates increased expression with improved SDB phenotypes, while purple colors indicates decreased expression with improved SDB phenotypes. Note that AHI effect estimates were oriented to match the directions of AvgO2 and MinO2.

Supplementary Figure 3. Spearman correlations between estimated log-fold changes in gene expression across SDB phenotypes and tissues in BMI adjusted analysis in MESA (top genes)

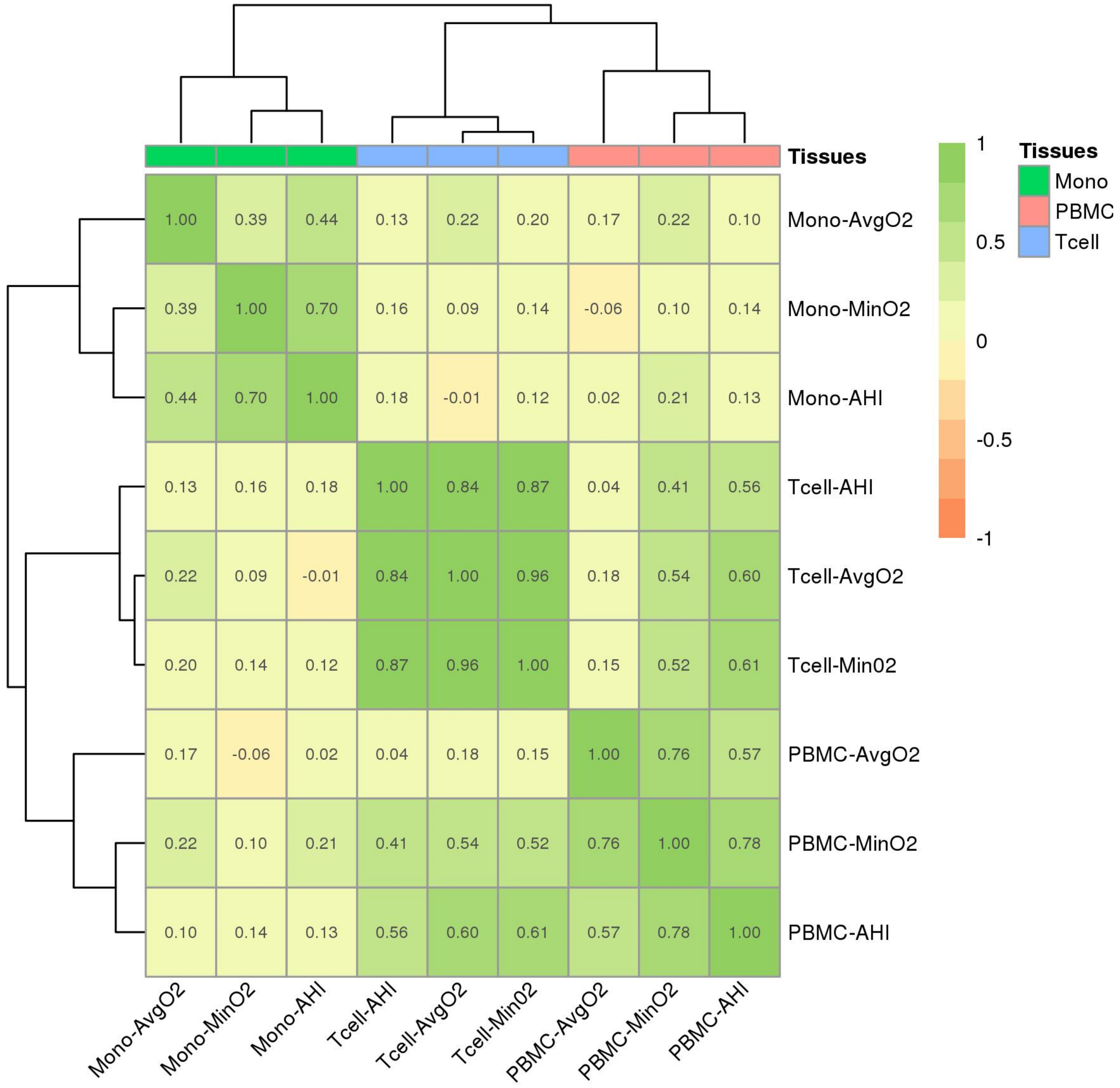

Heatmap illustrating the Spearman correlations of log-fold change of transcript expression by tissue type (monocytes, T-cells, PBMCs) and SDB phenotype (AvgO2, MinO2, AHI) in MESA. Correlations were computed over genes with FDR p<0.1. Color legend portrays Spearman R^2 (no/weak correlation = light yellow; complete/strong correlation = green). Estimated AHI effect sizes are flipped prior to computation of correlations so that they match the direction of MinO2 and AvgO2.

Supplementary Figure 4. Correlation between the SDB phenotypes

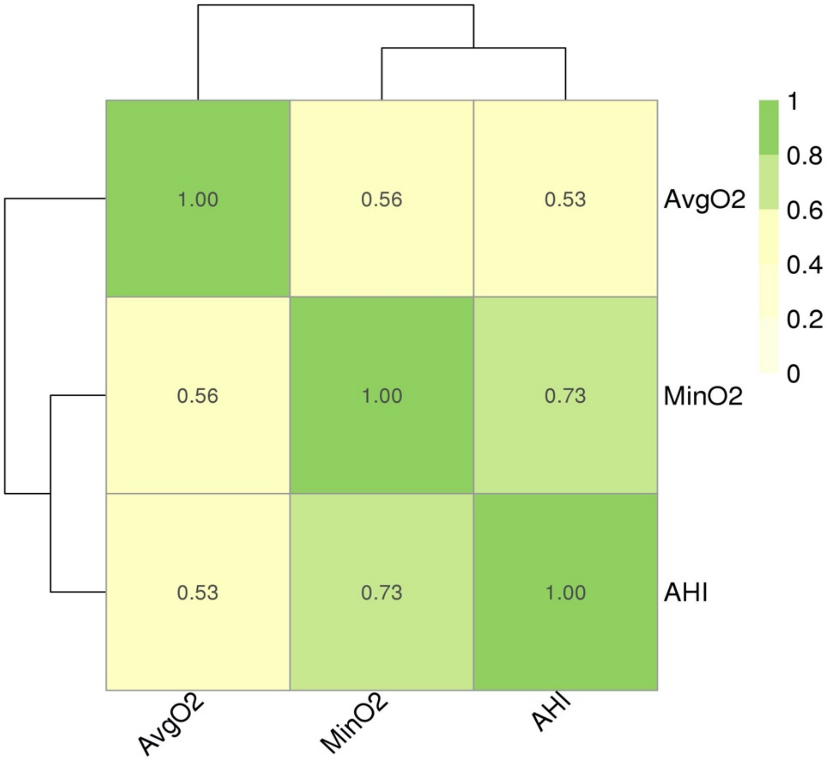

Heatmap illustrating the Spearman correlations SDB phenotype (AvgO2, MinO2, AHI). Color legend portrays Spearman R^2 (no/weak correlation = light yellow; complete/strong correlation = green).

Supplementary Figure 5. Spearman correlations between estimated log-fold changes in gene expression across SDB phenotypes and tissues in analysis without BMI adjustment (all genes).

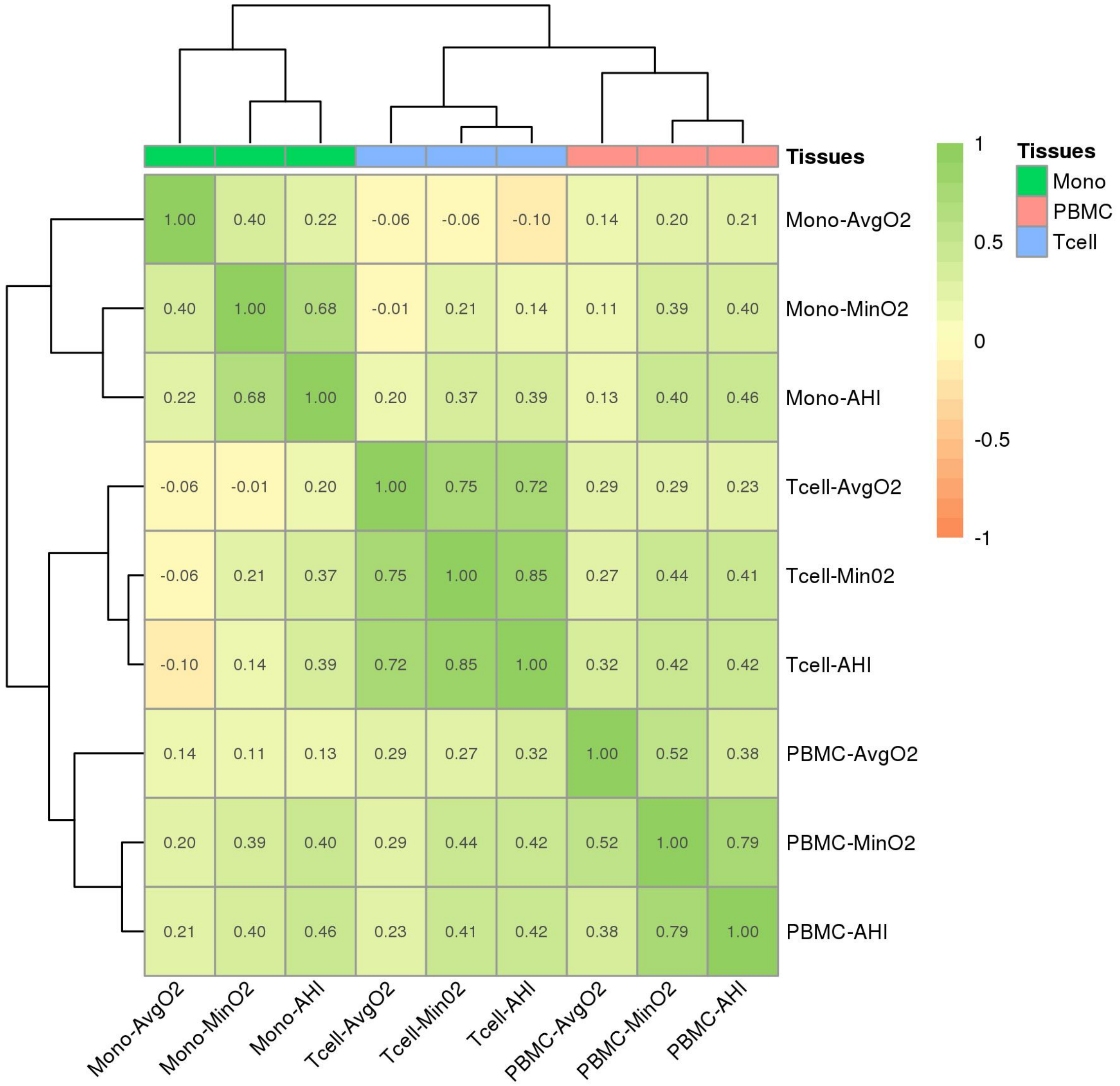

Heatmap illustrating the Spearman correlations of log-fold change of transcript expression by tissue type (monocytes, T-cells, PBMCs) and SDB phenotype (AvgO2, MinO2, AHI). Color legend portrays Spearman R^2 (no/weak correlation = light yellow; complete/strong correlation = green).

Supplementary Figure 6: Spearman correlations between estimated log-fold changes in gene expression across SDB phenotypes and tissues in analysis BMI adjustment (all genes).

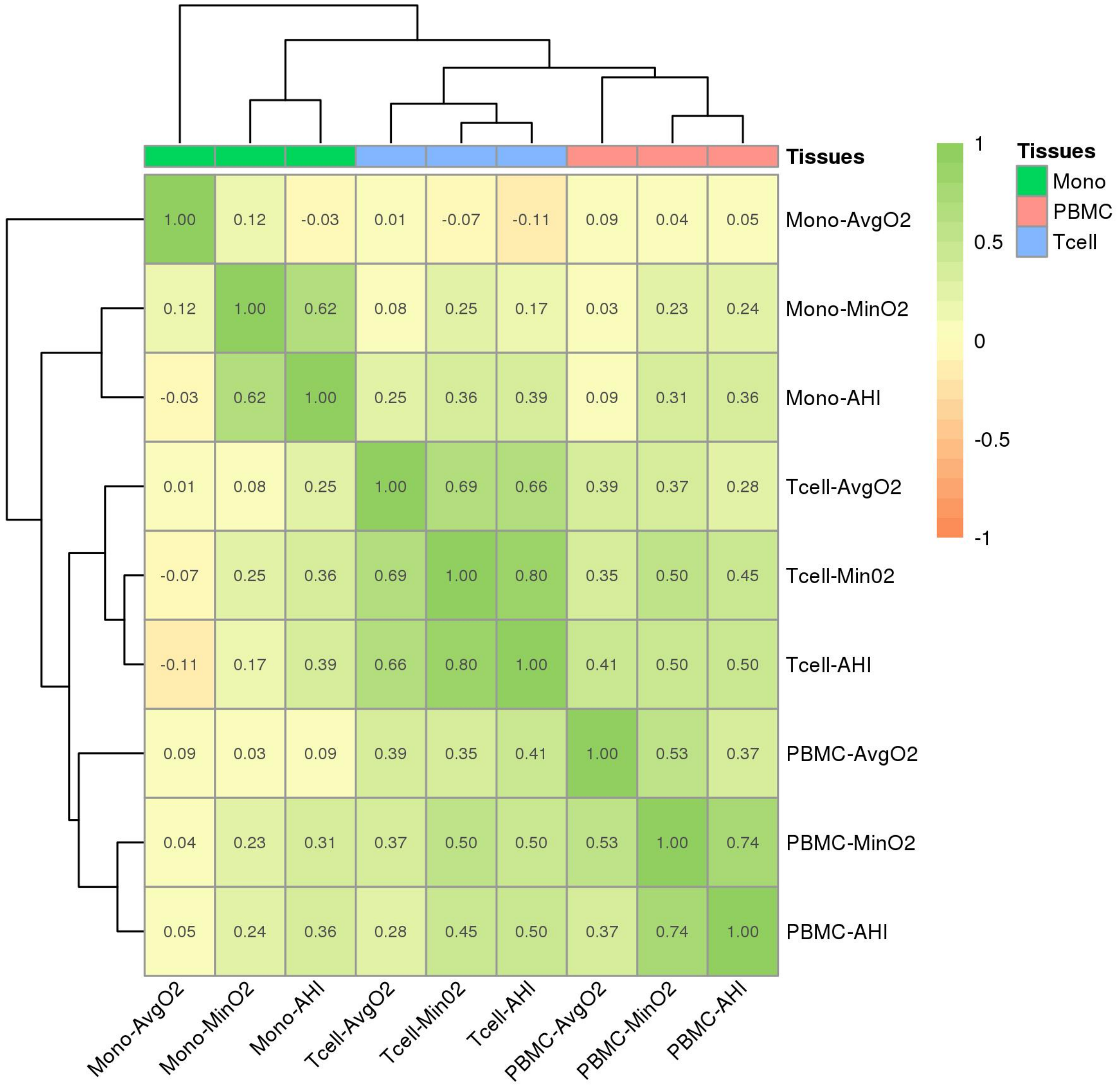

Heatmap illustrating the Spearman correlations of log-fold change of transcript expression by tissue type (monocytes, T-cells, PBMCs) and SDB phenotype (AvgO2, MinO2, AHI). Color legend portrays Spearman R^2 (no/weak correlation = light yellow; complete/strong correlation = green).

Supplementary Figure 7. Comparison of the associations between monocyte-based tPRSs and whole-blood gene expression in WHI

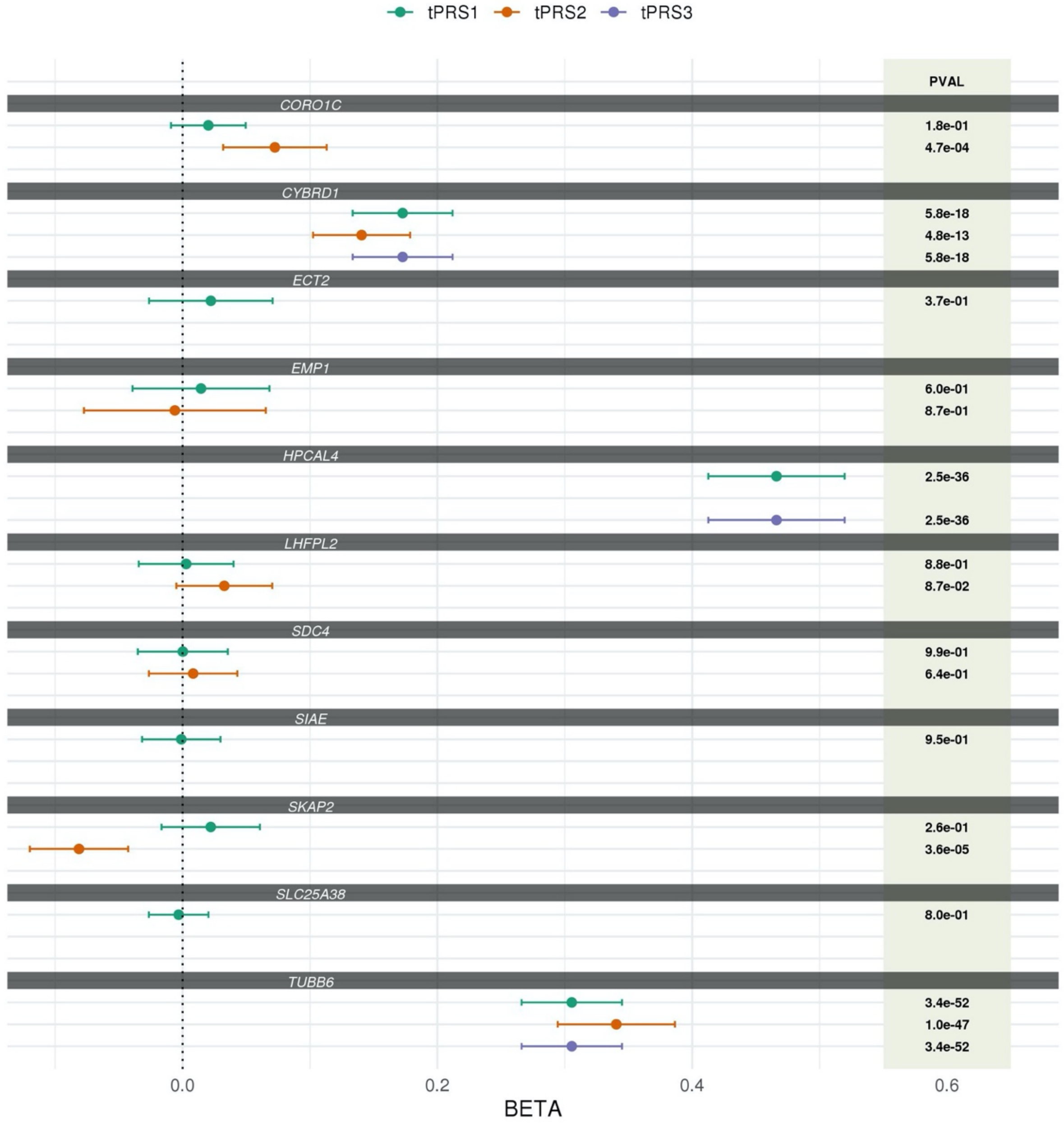

For each transcript associated with an SDB phenotype, the figure provides the estimated association effect, 95% confidence interval, and p-value based on the 1 degree-of-freedom Wald test, of tPRSs constructed in different approaches with whole-blood transcript expression in WHI. tPRS1 and tPRS3 were constructed using the clump and threshold approach implemented in PRSice2 using summary statistics from GWAS of transcript expression in monocytes in MESA, and with clumping guided by LD in MESA (the same individuals used for GWAS). tPRS1 allows for genome-wide SNPs, and tPRS3 focused on cis-eQTLs. tPRS2 is the prediXcan model (also using cis-eQLTs only). For tPRS1 and 3 we considered three p-value threshold ($5x10^{-8}$, 10^{-7}, and 10^{-6}), and the tPRS with smallest p-value is displayed. PRS associations were estimated in models adjusted for sex, age, study site, race/ethnic background, batch effects, and 11 ancestral principal components. The sample size was n=1,253 for all tPRS.

Supplementary Figure 8. MESA data flow across various measures in the 1st and 5th exam, and the sleep ancillary study.

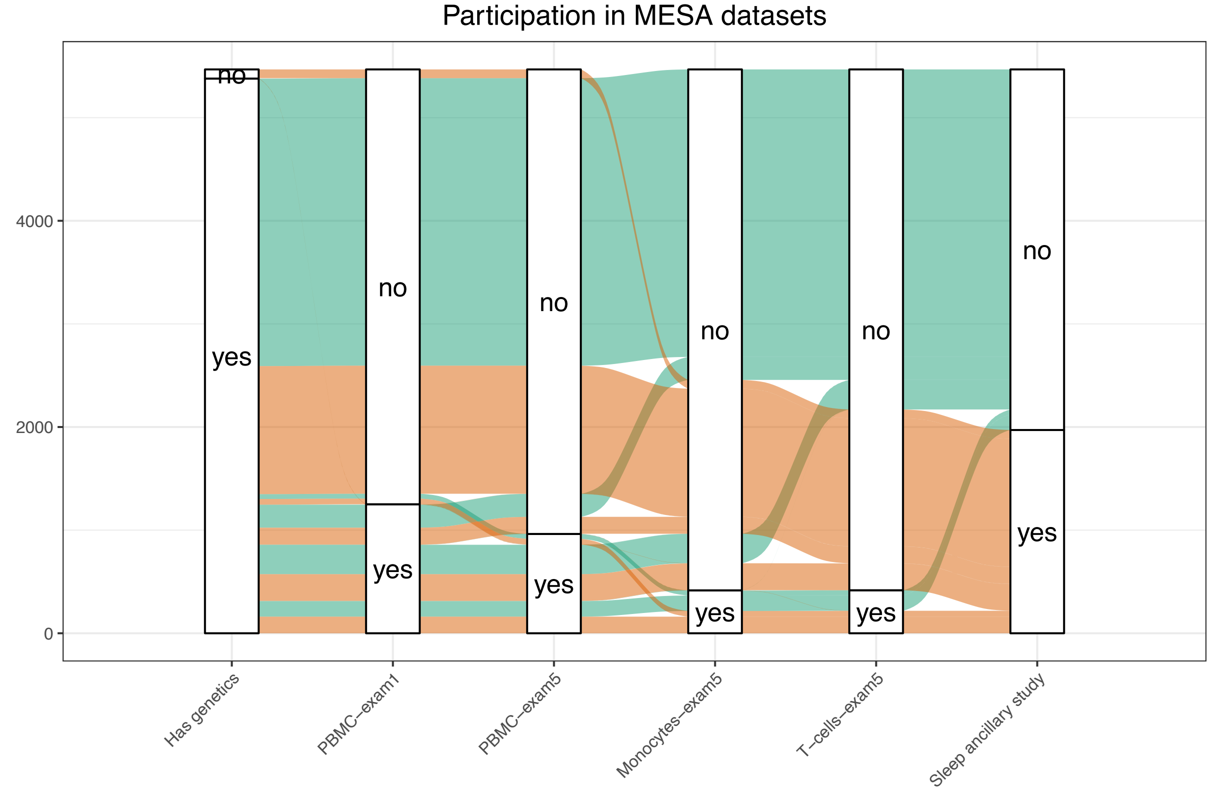

The figure focuses on n=5,468 individuals having at least one of the displayed measures.

Supplementary Figure 9. HCHS/SOL data flow across genotyping, metabolomics, and sleep data.

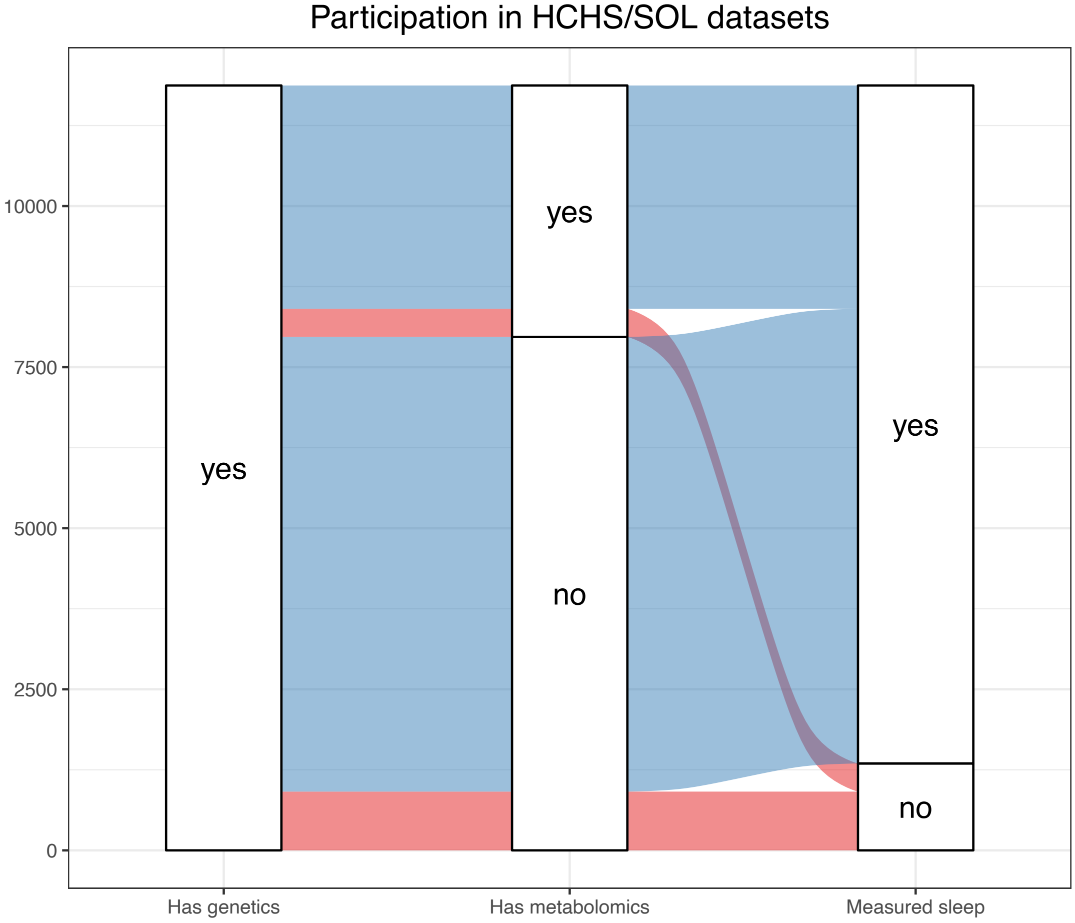

The figure focuses on n=11,872 with genetic data.

Supplementary References

1. Bild, D. E. *et al.* Multi-Ethnic Study of Atherosclerosis: objectives and design. *Am. J. Epidemiol.* **156,** 871–881 (2002).
2. Chen, X. *et al.* Racial/Ethnic Differences in Sleep Disturbances: The Multi-Ethnic Study of Atherosclerosis (MESA). *Sleep* **38,** 877–888 (2015).
3. Sorlie, P. D. *et al.* Design and implementation of the Hispanic Community Health Study/Study of Latinos. *Ann Epidemiol* **20,** 629–641 (2010).
4. Lavange, L. M. *et al.* Sample design and cohort selection in the Hispanic Community Health

Study/Study of Latinos. *Ann Epidemiol* **20,** 642–649 (2010).
5. Conomos, M. P. *et al.* Genetic diversity and association studies in US hispanic/latino populations: applications in the hispanic community health study/study of latinos. *Am. J. Hum. Genet.* **98,** 165–184 (2016).
6. Kowalski, M. H. *et al.* Use of >100,000 NHLBI Trans-Omics for Precision Medicine (TOPMed) Consortium whole genome sequences improves imputation quality and detection of rare variant associations in admixed African and Hispanic/Latino populations. *PLoS Genet.* **15,** e1008500 (2019).
7. Feofanova, E. V. *et al.* A Genome-wide Association Study Discovers 46 Loci of the Human Metabolome in the Hispanic Community Health Study/Study of Latinos. *Am. J. Hum. Genet.* **107,** 849–863 (2020).
8. Hays, J. *et al.* The Women's Health Initiative recruitment methods and results. *Ann Epidemiol* **13,** S18-77 (2003).
9. Paz, M. A. *et al.* Treatment efficacy of anti-hypertensive drugs in monotherapy or combination: ATOM systematic review and meta-analysis of randomized clinical trials according to PRISMA statement. *Medicine* **95,** e4071 (2016).
