# Supplementary material for: An integrated multi-omics analysis of sleep-disordered breathing traits implicates P2XR4 purinergic signaling
Source: Commun Biol. 2023 Jan 31;6:125. doi: 10.1038/s42003-023-04520-y (PMC9889381; doi:10.1038/s42003-023-04520-y)
Supplement: Supplementary file 3 — Description of Additional Supplementary Files [file 42003_2023_4520_MOESM3_ESM.pdf]

Description of Additional Supplementary Files

File name: Supplementary Data 1

Description: Characteristics of MESA participants in the sleep-disordered breathing analysis, grouped by tissue type.

File name: Supplementary Data 2

Description: Characteristics table of HCHS/SOL participants included in the sleep-disordered breathing and transcript PRS analysis.

File name: Supplementary Data 3

Description: Characteristics table of WHI RNA-Seq participants.

File name: Supplementary Data 4

Description: Differentially expressed transcripts (FDR p-value<0.1) associated with SDB phenotypes in MESA, without BMI adjustment.

File name: Supplementary Data 5

Description: Differentially expressed transcripts (FDR p-value<0.1) associated with multiple SDB phenotypes in MESA, without BMI adjustment.

File name: Supplementary Data 6

Description: Differentially expressed transcripts (FDR p-value<0.1) associated with SDB phenotypes in MESA adjusted for BMI.

File name: Supplementary Data 7

Description: Differentially expressed transcripts (FDR p-value<0.1) associated with multiple SDB phenotypes in MESA, adjusted for BMI.

File name: Supplementary Data 8

Description: Generalized and validated of tPRS with gene expression in WHI without adjusting for BMI (p-value < 0.017).

File name: Supplementary Data 9

Description: Generalized and validated of tPRS with gene expression in WHI adjusting for BMI (p-value < 0.017).

File name: Supplementary Data 10

Description: Association of tPRS with SDB phenotype in HCHS/SOL, without adjusting for BMI.

File name: Supplementary Data 11

Description: Association of transcript PRSs with sleep phenotype in HCHS/SOL, adjusted for BMI.

File name: Supplementary Data 12

Description: Association of tPRS with metabolites (FDR BH<0.05), unadjusted for BMI.

File name: Supplementary Data 13

Description: Association of tPRS with metabolites (FDR BH<0.05), adjusted for BMI < 0.05).

File name: Supplementary Data 14

Description: Association of SDB phenotypes with metabolites "connected" via transcript PRS in HCHS/SOL, unadjusted for BMI.

File name: Supplementary Data 15

Description: Association of SDB phenotypes with metabolites "connected" via transcript PRS in HCHS/SOL, adjusted for BMI.

File name: Supplementary Data 16
Description: The source data behind Figure 1 in the paper.
