# Supplementary material for: An integrated multi-omics analysis of sleep-disordered breathing traits implicates P2XR4 purinergic signaling
Source: Commun Biol. 2023 Jan 31;6:125. doi: 10.1038/s42003-023-04520-y (PMC9889381; doi:10.1038/s42003-023-04520-y)
Supplement: Supplementary file 6 — Reporting Summary [file 42003_2023_4520_MOESM6_ESM.pdf]

nature portfolio

Corresponding author(s): Dr. Tamar sofer

Last updated by author(s): Jan 13, 2023

Reporting Summary

Nature Portfolio wishes to improve the reproducibility of the work that we publish. This form provides structure for consistency and transparency in reporting. For further information on Nature Portfolio policies, see our Editorial Policies and the Editorial Policy Checklist.

Statistics

For all statistical analyses, confirm that the following items are present in the figure legend, table legend, main text, or Methods section.

n/a	Confirmed	
☐	☒	The exact sample size (*n*) for each experimental group/condition, given as a discrete number and unit of measurement
☐	☒	A statement on whether measurements were taken from distinct samples or whether the same sample was measured repeatedly
☐	☒	The statistical test(s) used AND whether they are one- or two-sided<br>*Only common tests should be described solely by name; describe more complex techniques in the Methods section.*
☐	☒	A description of all covariates tested
☐	☒	A description of any assumptions or corrections, such as tests of normality and adjustment for multiple comparisons
☐	☒	A full description of the statistical parameters including central tendency (e.g. means) or other basic estimates (e.g. regression coefficient) AND variation (e.g. standard deviation) or associated estimates of uncertainty (e.g. confidence intervals)
☐	☒	For null hypothesis testing, the test statistic (e.g. *F*, *t*, *r*) with confidence intervals, effect sizes, degrees of freedom and *P* value noted<br>*Give P values as exact values whenever suitable.*
☒	☐	For Bayesian analysis, information on the choice of priors and Markov chain Monte Carlo settings
☒	☐	For hierarchical and complex designs, identification of the appropriate level for tests and full reporting of outcomes
☐	☒	Estimates of effect sizes (e.g. Cohen's *d*, Pearson's *r*), indicating how they were calculated

Our web collection on statistics for biologists contains articles on many of the points above.

Software and code

Policy information about availability of computer code

Data collection	No software was used.
Data analysis	The following softwares were used: R/4.0.3,PRSice2 v2.3.3 , PLINK v1.9, PLINKv.2.0,Python 3.6.13, MetaXcan, GENESIS v2.16.1,Survey v4.0, Olivia R package.<br>Custom code can be found at : https://github.com/nkurniansyah/SDB_Multi_Omics

For manuscripts utilizing custom algorithms or software that are central to the research but not yet described in published literature, software must be made available to editors and reviewers. We strongly encourage code deposition in a community repository (e.g. GitHub). See the Nature Portfolio guidelines for submitting code & software for further information.

Data

Policy information about availability of data

All manuscripts must include a data availability statement. This statement should provide the following information, where applicable:

- Accession codes, unique identifiers, or web links for publicly available datasets
- A description of any restrictions on data availability
- For clinical datasets or third party data, please ensure that the statement adheres to our policy

MESA, HCHS/SOL and WHI data are available through application to dbGaP according to the study specific accessions. MESA phenotypes are available in: “phs000209 [https://www.ncbi.nlm.nih.gov/projects/gap/cgi-bin/study.cgi?study_id=phs000209.v13.p3]”; WHI phenotypes: “phs000200 [https://

www.ncbi.nlm.nih.gov/projects/gap/cgi-bin/study.cgi?study_id=phs000200.v12.p3]"; and HCHS/SOL phenotypes: "phs000810 [https://www.ncbi.nlm.nih.gov/projects/gap/cgi-bin/study.cgi?study_id=phs000810.v1.p1]". HCHS/SOL genotyping data: "phs000880[https://www.ncbi.nlm.nih.gov/projects/gap/cgi-bin/study.cgi?study_id=phs000880.v1.p1]". MESA and WHI RNA-seq data has been deposited and will become available through the TOPMed according to the study specific accessions; MESA: "phs001416 [https://www.ncbi.nlm.nih.gov/projects/gap/cgi-bin/study.cgi?study_id=phs001416.v2.p1]"; WHI: "phs001237 [https://www.ncbi.nlm.nih.gov/projects/gap/cgi-bin/study.cgi?study_id=phs001237.v3.p1]". HCHS/SOL metabolomics data are available via data use agreement with the HCHS/SOL Data Coordinating Center at the University of North Carolina at Chapel Hill, see collaborators website: https://sites.cscc.unc.edu/hchs/. Data needed to construct the tPRS are publicly available on the repository https://github.com/nkurniansyah/SDB_Multi_Omics, https://doi.org/10.5281/zenodo.7320074. Complete summary statistics from SDB traits association analyses with RNA-seq across cell types are provided in the same zenodo repository.

Human research participants

Policy information about studies involving human research participants and Sex and Gender in Research.

Reporting on sex and gender	All analyses were adjusted for biological sex, where sex was validated using chromosomal checks in all datasets. With the exception that one of the datasets, WHI, includes only women/female participants. The number of male and female participants are reporting in Supplementary Tables 1, 2, and 3.
Population characteristics	See Supplementary Tables 1-3. Briefly, All participants in our studies (MESA, WHI, and HCHS/SOL) are a multi-ethnic sample, and the races and ethnicities are self-reported. In MESA, we used age at exam 1 and exam 5, with 60 and 69 years old on average; In WHI, we used age at the Long-Life Study exam when RNA was extracted with 80 years old on average; In HCHS/SOL, the average of age is 46 years during the baseline exam.
Recruitment	-MESA is a longitudinal cohort study (27), established in 2000, that prospectively collected risk factors for development of subclinical and clinical cardiovascular disease among participants in six field centers across the United States (Baltimore City and Baltimore County, MD; Chicago, IL; Forsyth County, NC; Los Angeles County, CA; Northern Manhattan and the Bronx, NY; and St. Paul, MN). The 1st and 5th MESA exams took place between 2000-2002, and 2010-2012, respectively, and whole blood was drawn from participants in both exams. For about 1,400 participants, blood was used later for RNA extraction and/or proteomics in at least one of the exams. In addition, a sleep study ancillary to MESA occurred shortly after MESA exam 5 during 2010-2013. Sleep study participants underwent single night in-home polysomnography (Compumedics Somte Systems, Abbotsville, Australia, AU), as previously described (28). The number of individuals with each type of data and at each time point (exam 1 and exam 5) varies.<br>-The WHI is a prospective national health study focused on identifying optimal strategies for pre-venting chronic diseases that are the major causes of death and disability in postmenopausal women [3]. The WHI initially recruited 161,808 women between 1993 and 1997 with the goal of including a socio-demographically diverse population with racial/ethnic minority groups propor-tionate to the total minority population of US women aged 50-79 years. The WHI consists of two major parts: a set of randomized Clinical Trials and an Observational Study. The WHI Clinical Trials (CT; N=68,132) includes three overlapping components, each a randomized controlled comparison: the Hormone Therapy Trials (HT), Dietary Modification Trial, and Calcium and Vita-min D Trial. A parallel prospective observational study (OS; N = 93,676) examined biomarkers and risk factors associated with various chronic diseases. While the HT trials ended in the mid-2000s, active follow up of the WHI-CT and WHI-OS cohorts has continued for over 25 years with the accumulation of large numbers of diverse clinical outcomes, risk factor measurements, med-ication use, and many other types of data.<br>A total of 11,071 WHI participants have whole-genome sequencing data via TOPMed, and 1,274 of these participants have RNA-seq measured in venous blood via TOPMed.<br>- The HCHS/SOL is a longitudinal cohort study of U.S. Hispanics/Latinos (30,31) recruited from four geographic regions: Bronx NY, Chicago IL, Miami FL, and San Diego CA. The HCHS/SOL baseline exam occurred on 2008-2011, where 16,415 participants were enrolled via multi-stage probability sampling. HCHS/SOL individuals who consented further participated in an in-home sleep study, using a validated type 3 home sleep apnea test recording airflow (via nasal pressure), oximetry, position, and snoring (ARES Unicorder 5.2; B-Alert). Genetic data were measured and imputed to the TOPMed freeze 5b reference panel as previously described, for individuals who consented at baseline and Metabolomic data were also measured for n=~4,000 individuals selected at random out of those with genetic data.
Ethics oversight	- All MESA participants provided written informed consent, and the study was approved by the Institutional Review Boards at The Lundquist Institute (formerly Los Angeles BioMedical Research Institute) at Harbor-UCLA Medical Center, University of Washington, Wake Forest School of Medicine, Northwestern University, University of Minnesota, Columbia University, and Johns Hopkins University.<br>- The HCHS/SOL was approved by the institutional review boards (IRBs) at each field center, where all participants gave written informed consent, and by the Non-Biomedical IRB at the University of North Carolina at Chapel Hill, to the HCHS/SOL Data Coordinating Center. All IRBs approving the study are: Non-Biomedical IRB at the University of North Carolina at Chapel Hill. Chapel Hill, NC; Einstein IRB at the Albert Einstein College of Medicine of Yeshiva University. Bronx, NY; IRB at Office for the Protection of Research Subjects (OPRS), University of Illinois at Chicago. Chicago, IL; Human Subject Research Office, University of Miami. Miami, FL; Institutional Review Board of San Diego State University, San Diego, CA.<br>-All WHI participants provided informed consent and the study was approved by the IRB of the Fred Hutchinson Cancer Research Center.

Note that full information on the approval of the study protocol must also be provided in the manuscript.

Field-specific reporting

Please select the one below that is the best fit for your research. If you are not sure, read the appropriate sections before making your selection.

☒ Life sciences ☐ Behavioural & social sciences ☐ Ecological, evolutionary & environmental sciences

For a reference copy of the document with all sections, see nature.com/documents/nr-reporting-summary-flat.pdf

Life sciences study design

All studies must disclose on these points even when the disclosure is negative.

Sample size	-The sample size in MESA was 1779 participants who have RNA-seq data and WGS to construct tPRS.<br>-To validated the tPRS , We used WHI with total sample was 1274 who have RNA-Seq and WGS was 1274.<br>-Total Sample was used in HCHS/SOL metabolomics and genotype data were 3968 and 12689.
Data exclusions	No exclusions: all individuals from the participating studies with phenotype values and appropriate consent were included.
Replication	The association of tPRS that were developed in MESA were replicated for associations with RNAseq in WHI. Associations of sleep phenotypes with RNAseq discovered in MESA were then evaluated in HCHS/SOL with tPRS of RNAseq measures acting as instrumental variables for the RNAseq.
Randomization	There was no randomization because the data do not represent a trial, but rather an observational study.
Blinding	Because there was no trial/experiment, there was no blinding. We analyzed observational data

Reporting for specific materials, systems and methods

We require information from authors about some types of materials, experimental systems and methods used in many studies. Here, indicate whether each material, system or method listed is relevant to your study. If you are not sure if a list item applies to your research, read the appropriate section before selecting a response.

Materials & experimental systems

n/a	Involved in the study	
☒	☐	Antibodies
☒	☐	Eukaryotic cell lines
☒	☐	Palaeontology and archaeology
☒	☐	Animals and other organisms
☒	☐	Clinical data
☒	☐	Dual use research of concern

Methods

n/a	Involved in the study	
☒	☐	ChIP-seq
☒	☐	Flow cytometry
☒	☐	MRI-based neuroimaging
